# Microwave Imaging and Sensing Techniques for Breast Cancer Detection

**DOI:** 10.3390/mi14071462

**Published:** 2023-07-21

**Authors:** Lulu Wang

**Affiliations:** Biomedical Device Innovation Center, Shenzhen Technology University, Shenzhen 518118, China; lwang381@hotmail.com or wanglulu@sztu.edu.cn

**Keywords:** microwave imaging, breast imaging, microwave antenna, antenna array, breast cancer

## Abstract

Medical imaging techniques, including X-ray mammography, ultrasound, and magnetic resonance imaging, play a crucial role in the timely identification and monitoring of breast cancer. However, these conventional imaging modalities have their limitations, and there is a need for a more accurate and sensitive alternative. Microwave imaging has emerged as a promising technique for breast cancer detection due to its non-ionizing, non-invasive, and cost-effective nature. Recent advancements in microwave imaging and sensing techniques have opened up new possibilities for the early diagnosis and treatment of breast cancer. By combining microwave sensing with machine learning techniques, microwave imaging approaches can rapidly and affordably identify and classify breast tumors. This manuscript provides a comprehensive overview of the latest developments in microwave imaging and sensing techniques for the early detection of breast cancer. It discusses the principles and applications of microwave imaging and highlights its advantages over conventional imaging modalities. The manuscript also delves into integrating machine learning algorithms to enhance the accuracy and efficiency of microwave imaging in breast cancer detection.

## 1. Introduction

Breast cancer is the leading cause of female cancer-related death worldwide [1]. Approximately 13% of females are at risk of breast cancer in the United States [2]. Early detection is crucial for successful treatment, but the current imaging techniques have limitations. Mammography, the most widely used tool for breast cancer screening, cannot be utilized for pregnant women or those with dense breasts [3]. Ultrasound imaging, on the other hand, produces low-quality images that may not accurately identify cancerous cells [4]. Magnetic resonance imaging (MRI) is more effective in detecting breast cancer, but it is not only expensive but also uncomfortable for patients [5]. Given these limitations, researchers have been exploring alternative methods for breast cancer diagnosis, and microwave breast imaging (MBI) has shown promise.

**Figure 1:**
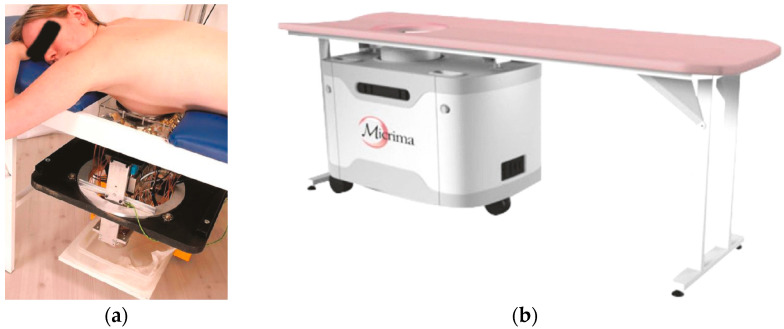
Microwave radar-based breast imaging systems [3] (**a**) at Bristol University and (**b**) from Micrima Ltd. Image from the article published under an open-access Creative Common CC BY license.

**Figure 2:**
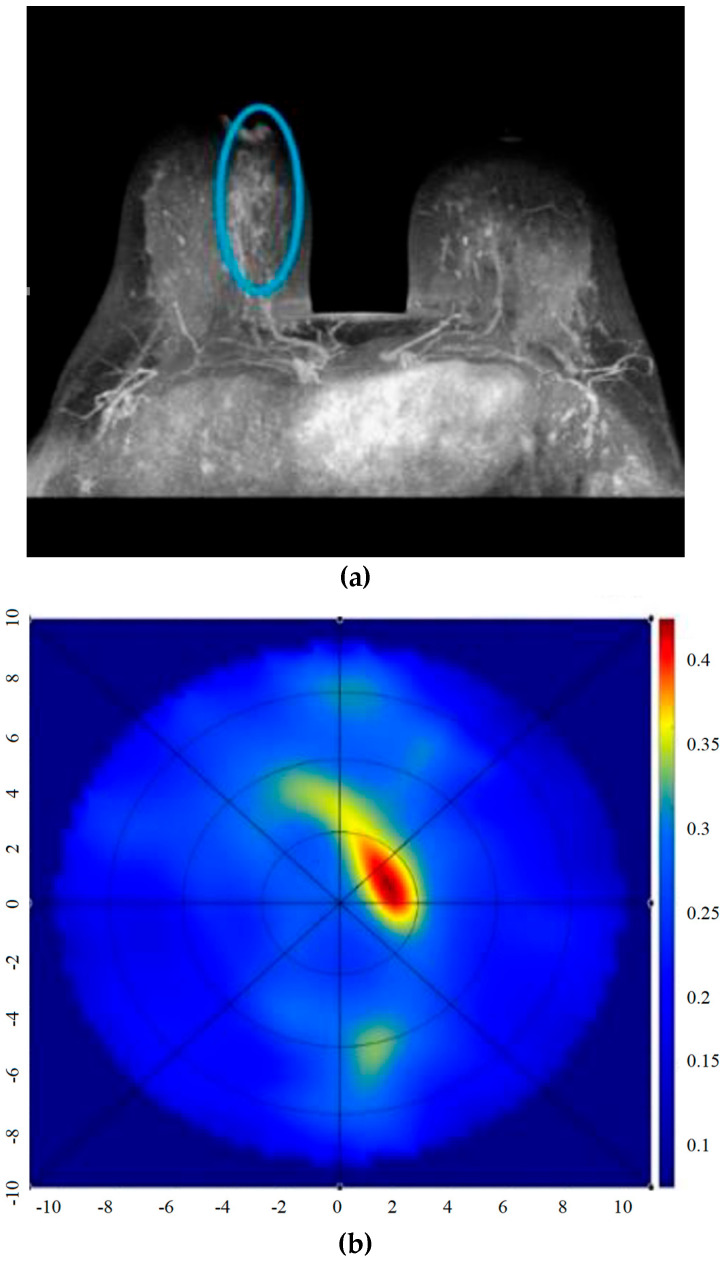

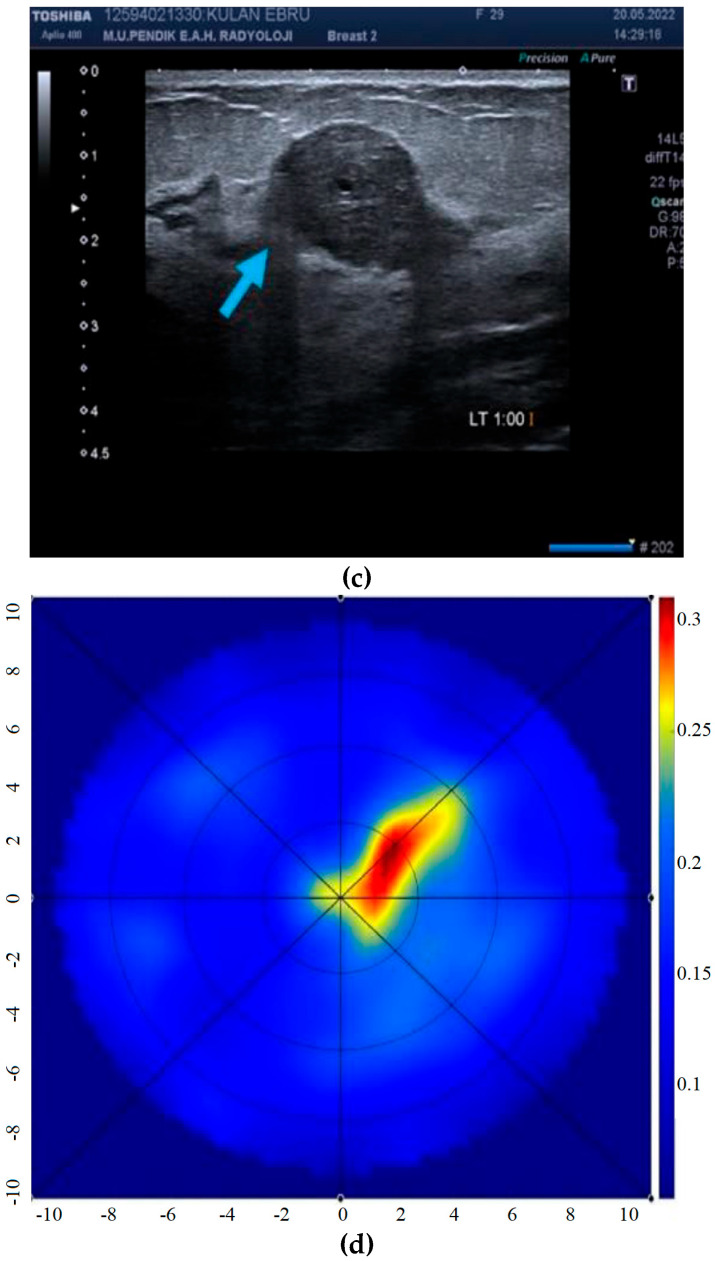
Comparison of clinical images [6]. (**a**) MRI image of the malignant lesion (blue circle) in a non-dense breast; (**b**) microwave image of the malignant lesion (high-contrast region) inside the scanning domain (blue circle) at the same location as reported via MRI; (**c**) ultrasound image of the benign lesion in the breast of unknown density (blue arrow); (**d**) microwave image of the benign lesion (high-contrast region), inside the scanning domain (blue circle), at the same location as reported via ultrasound. Images from articles published under an open-access Creative Common CC BY license.

MBI is non-invasive, meaning it does not require invasive procedures and does not utilize ionizing radiation, making it safe for patients. Additionally, it is cost-effective, making it a viable option for widespread use [6]. As shown in Figure 1, MBI works by analyzing and differentiating the changes in backscattered signals caused by the differences in dielectric properties between cancerous and normal cells [7]. This enables the identification of malignant cells, which is crucial for an accurate diagnosis. Although holographic microwave imaging (HMI) algorithms have shown potential for breast cancer detection, further development, and validation are necessary before these techniques can be implemented in clinical trials [8,9,10,11,12,13]. Some MBI systems have already reached the clinical trials stage, as shown in Figure 2 [14,15]. However, these systems have limitations, such as low-resolution images, coupling noises between antennas, and system complexity.

**Figure 3:**
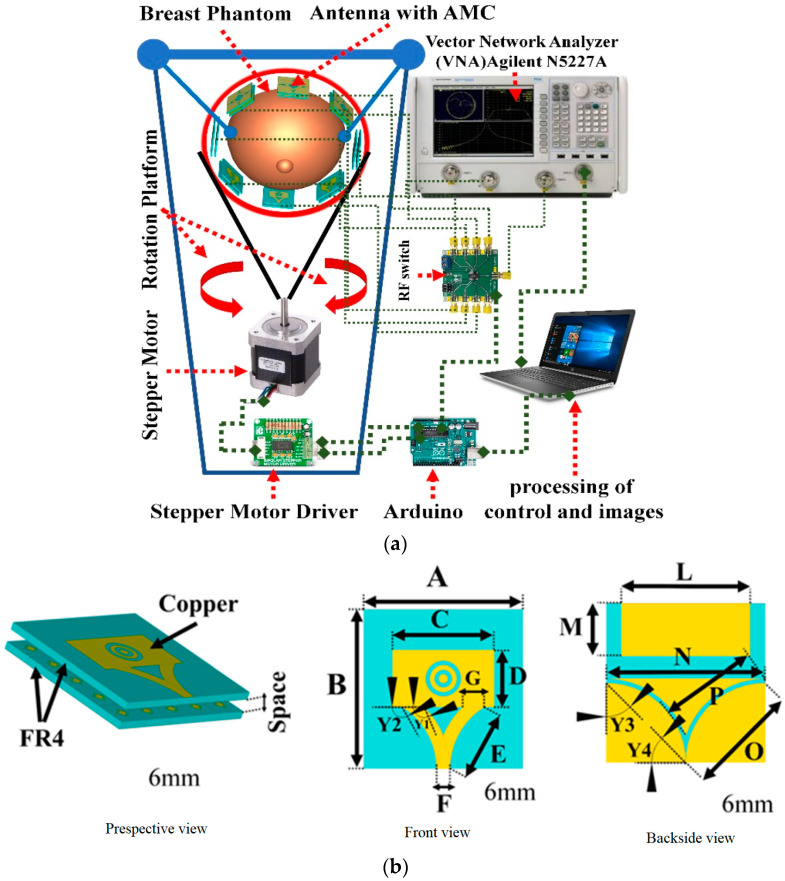
(**a**) An example of a breast imaging system consisting of several microstrip patch antennas; (**b**) an example of a microstrip patch antenna [16]—images from articles published under an open-access Creative Common CC BY license.

In the MBI system, the antenna plays a vital role as it transmits microwave signals into the breast and measures the backscattered signals (see Figure 3) [16]. To make MBI portable and affordable, custom sensors are required. Ultra-wideband (UWB) patch antennas are commonly used in MBI applications. However, there is room for improvement in their radiation capacity, gain, and bandwidth [17]. Recently, there have been advancements in the development of custom antennas using metamaterial (MTM), metasurface (MTS), and artificial magnetic conductor (AMC) technologies [16,17,18,19,20]. These technologies present opportunities for the sensitivity and accuracy of MBI systems. They have improved the overall performance of MBI systems and significantly increased detection accuracy [17,21,22]. However, despite these advancements, there are still limitations to these antennas, and further research and development are needed to overcome these limitations and fully harness the potential of custom antennas for MBI applications.

Researchers globally have made significant progress in advancing the development of MBI technologies. These technologies can potentially revolutionize breast cancer diagnosis by providing a non-invasive, radiation-free, and cost-effective alternative to traditional imaging techniques such as mammography. However, despite the progress made, further research and development are needed to establish connections between research labs, industrial partners, and patients who can benefit from these technologies. Collaboration between these stakeholders is essential for translating research findings into practical clinical applications. One of the critical technical challenges that researchers must address is enhancing imaging algorithms to interpret microwave signals obtained during the imaging process accurately. These algorithms play a crucial role in reconstructing high-resolution images of breast tissue and detecting abnormalities. Improving the accuracy of these algorithms will enable healthcare professionals to make more reliable and precise diagnoses. In addition to algorithm development, researchers must also focus on developing high-sensitivity acquisition hardware. The hardware used in MBI systems must be capable of capturing weak microwave signals emitted by breast tissue. By enhancing the sensitivity of the hardware, researchers can improve the signal-to-noise ratio and enhance the overall performance of MBI systems. Overcoming these technical challenges is crucial for improving the performance of MBI systems and increasing their reliability and effectiveness in detecting breast abnormalities. By addressing these challenges, researchers can pave the way for the widespread adoption of MBI technologies in breast cancer diagnosis.

This manuscript presents state-of-the-art microwave imaging and sensing methods for early breast cancer diagnosis. The manuscript also discusses the dielectric properties of breast tissues and how they can be utilized for microwave imaging. Furthermore, the manuscript reviews recently developed MBI techniques, including deep learning-based approaches. These approaches leverage advanced machine learning algorithms to improve the accuracy and sensitivity of MBI systems. The paper is structured as follows. Section 1 introduces the background of this study. Section 2 provides an overview of breast imaging techniques, and Section 3 discusses the dielectric properties of breast tissues. Section 4 reviews recently developed microwave breast imaging techniques, including deep learning-based approaches. Section 5 presents microwave sensing techniques for breast cancer detection, while Section 6 addresses the challenges and prospects of microwave imaging and sensing techniques, and Section 7 concludes this study.

## 2. Breast Imaging Techniques

Conventional medical imaging modalities, such as X-ray mammography, ultrasound, MRI, CT, and positron emission tomography (PET), play a pivotal role in detecting breast cancer. X-ray mammography is commonly employed as the first line of defense due to its simple operation, high resolution, and high repeatability. However, it may not be a suitable option for all women, particularly those with dense breast tissue or who are pregnant [3]. Ultrasound imaging is a safe alternative and more convenient for high-risk patients. Still, it may not reveal breast lesions in adipose tissue and has a relatively low detection rate for malignant tumors [4]. MRI is a sensitive imaging technique that is particularly effective in detecting tumors in dense breast tissue but has limitations due to its cost, magnetic field exposure, and the noise generated during the procedure [5]. Breast CT examination provides a detailed view of breast tissue but is limited by its high radiation exposure and cost [23]. PET imaging is a powerful method for detecting breast cancer. Still, it has limitations due to its unsuitability for early stage tumor detection, the potential false positives in young patients, and the need for simultaneous use with CT imaging and its associated radiation exposure and expense [24].

MBI has been proposed as a complementary modality for early breast cancer diagnosis to overcome some limitations of conventional medical imaging modalities. Researchers aim to integrate this innovative imaging technique into routine clinical practice, providing patients with an accurate and reliable tool for detecting breast abnormalities at an early stage. Despite the significant progress in advancing MBI technologies, further research and development are necessary to establish connections between research labs, industrial partners, and patients who can benefit from these technologies.

## 3. Dielectric Properties of Breast Tissues

Microwave imaging (MWI) leverages electromagnetic (EM) waves ranging from 300 MHz to 30 GHz, penetrating the breast tissue. As these waves traverse the tissue, they interact with its dielectric properties, including permittivity ($\varepsilon_{r}$, the capacity to store electrical energy) and conductivity (σ, the ability to conduct electrical energy). Normal and malignant tumor tissues exhibit distinct dielectric properties, causing EM waves to scatter and reflect in each tissue type at varying degrees. This scattering and reflection serve as the foundation for the microwave imaging of breast tumors.

**Figure 4:**
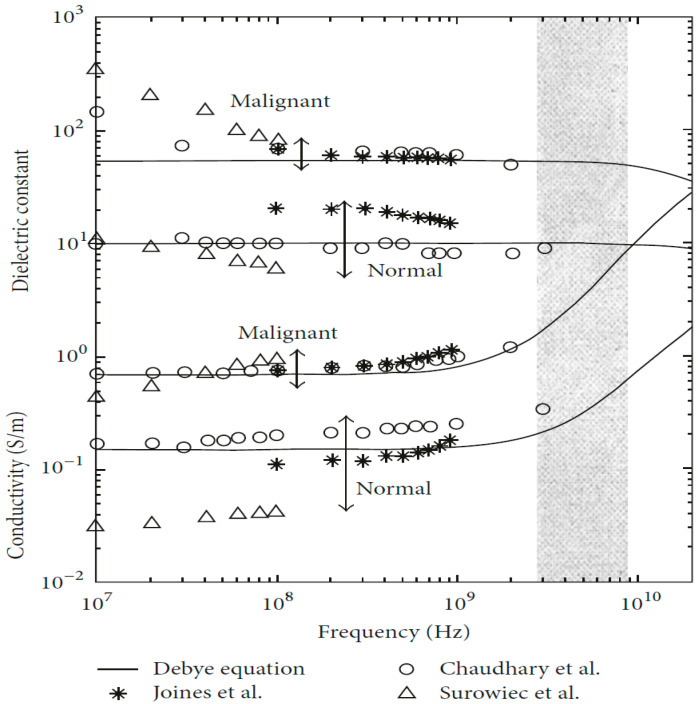
Dielectric property data for normal and malignant breast tissue [3]. Image from articles published under an open-access Creative Common CC BY license.

As shown in Figure 4, the dielectric properties of breast tissue change with working frequencies, and it is a nonlinear dependency [25]. The Debye and Cole–Cole models are commonly used to simulate biological tissues. The Debye model can be defined as follows [26]:

(1) $$\varepsilon_{r}=\varepsilon_{\infty }+\frac{\varepsilon_{s}+\varepsilon_{\infty }}{1+j\omega \tau }-j\frac{\sigma }{\omega \varepsilon_{o}}$$

where $\varepsilon_{\infty }$ denotes the permittivity, and its value strongly corresponds to the water content of the tissue, $\varepsilon_{s}$ represents the static permittivity, and τ denotes the relaxation time.

The Cole–Cole model can represent the complex dielectric constant of biological tissues [27].

(2) $$\varepsilon^{*}(\omega )=\varepsilon_{\infty }+\frac{\varepsilon_{s}+\varepsilon_{\infty }}{1+{\left(j\omega \tau \right)}^{1-\alpha }}$$

where $\varepsilon^{*}$ denotes the complex dielectric, $\varepsilon_{s}$ means the static frequency constant, $\varepsilon_{\infty }$ represents infinite frequency constant, ω means the angular frequency, and τ denotes the time constant. The exponent parameter α (0 < α < 1) represents different spectral shapes. When α=0, the Cole–Cole model becomes the Debye model. When α>0, the relaxation time is increased.

The following empirical model can represent the relationship between the dielectric parameters and the moisture content model [28].

(3) $$\varepsilon_{r}^{\prime }=1.71f^{1.3}+\frac{\varepsilon_{s}-4}{1+{\left(f/25\right)}^{2}}$$

(4) $$\sigma =1.35\sigma_{0.1}f^{0.13}+0.00222f^{2}\left[\frac{\varepsilon_{s}-4}{1+{\left(f/25\right)}^{2}}\right]$$

where f is the frequency, $\sigma_{0.1}=0.05$, and $\varepsilon_{s}=8.5$.

The dielectric properties of breast tissue exhibit a nonlinear dependence on working frequencies, which is commonly simulated using Debye or Cole–Cole models. These models consider the tissue’s water content and relaxation time. Experimental studies have shown that normal and malignant breast tissues have different electromagnetic responses due to differences in water and salt content [29,30]. Researchers have extensively studied the dielectric properties of biological tissues under the irradiation of electromagnetic waves with different frequencies, with findings indicating a significant difference in the dielectric properties of normal and tumor tissues [31,32]. Chaudhary et al. [33] discovered a substantial difference in the dielectric properties of normal and tumor tissues over the frequency range of 3–100 MHz. Joines et al. [34] replicated a similar study and confirmed Chaudhary’s findings. Gabriel et al. [29,30] reported their research findings on the characterization of biological tissues over the frequency range of 0.01 GHz to 20 GHz.

Furthermore, tissue at the infiltrating edge of the tumor has been found to have increased dielectric properties. Surowiec et al. [35] observed that the tissue at the infiltrating edge of the tumor had increased dielectric properties. Lazebnik et al. [31,32] studied the characterization of the dielectric properties of normal, malignant, and benign breast tissues over the frequency range of 0.5 GHz to 20 GHz. Halter et al. [36] conducted a similar measurement study on in vivo tissue, investigating the dielectric properties of breast tissues with and without tumor presence. Results showed negligible effects on the dielectric properties of tissues between excision and measurement.

Abas et al. [37] reported that the dielectric properties of tissues change significantly in the first few seconds after tissue excision. The authors investigated these effects based on ex vivo breast tissue measurement results. It is important to note that tissue dielectric properties can change significantly in the first few seconds after tissue excision due to temperature and water content changes.

Researchers have also developed breast phantoms based on measured dielectric properties of human breast tissue in the frequency range of 0.5–50 GHz, which have been tested using microwave imaging systems. Martellosio et al. [38] investigated the dielectric properties of breast tissues for the frequency range from 0.5 to 50 GHz. They employed Cole–Cole models for analyzing normal and tumorous tissues based on experimental measurements of 222 tissue samples from 53 patients aged 28 to 85. More recently, Meo et al. [39] developed three breast phantoms according to the dielectric properties of human breast ex vivo tissues in the frequency range of 0.5–50 GHz. The developed breast phantoms were tested using a microwave imaging system.

## 4. Microwave Breast Imaging Techniques

Microwave breast imaging (MBI) techniques can be classified into passive, hybrid, and active methods. Each method has its advantages and limitations. Passive methods rely solely on the radiation emitted by the body to create an image of the breast tissue. This approach does not require any external radiation sources. Passive methods are non-invasive but may have lower sensitivity. Hybrid microwave imaging combines microwave imaging with other imaging modalities such as ultrasound, MRI, or optical imaging. This combination of imaging techniques provides clinicians with more comprehensive and detailed information for accurate breast cancer diagnosis. Active methods utilize an external source of microwave radiation to probe the breast tissue. Active methods are more sensitive but need additional external radiation sources. Active MBI techniques such as microwave tomography (MT) and radar-based techniques have shown promise in accurately diagnosing breast cancer lesions. Radar-based methods use microwave radar to scan breast tissue in a non-invasive manner.

### 4.1. Microwave Tomography

Microwave tomography (MT) is an advanced imaging technique that uses microwave frequencies to create detailed cross-sectional images of the breast. It takes advantage of the different electrical properties of different types of tissues to accurately detect and visualize abnormalities, particularly breast cancer. Various methods are commonly employed in MT to process the collected data and solve inverse problems. Gradient-based approaches (such as conjugate gradient least squares and Landweber) and global techniques (such as genetic algorithm and particle swarm optimization) are utilized [40,41,42,43]. Several image reconstruction algorithms have been developed and applied in MT for detecting breast cancer, enhancing the accuracy and reliability of the imaging technique [44,45,46,47,48].

**Figure 5:**
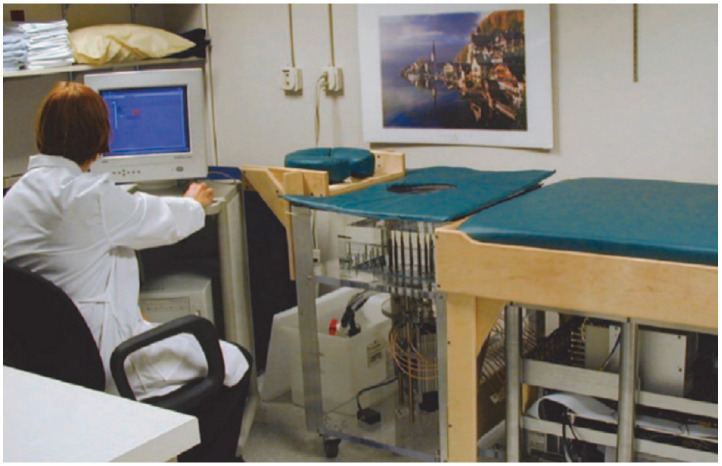
Microwave tomography at Dartmouth College [49]. Image from articles published under an open-access Creative Common CC BY license.

Meaney et al. [49] developed the first 2D MT clinical prototype (see Figure 5) for breast imaging, consisting of a cylindrical array of 16 monopole antennas that function as transceivers, operating at frequencies ranging from 300 to 1000 MHz. However, due to its complexity, this prototype is time-consuming and requires heavy computational work. To overcome these limitations, the same research group developed a 3D prototype for breast cancer detection using the 3D finite element modeling (FEM) method [50]. This new system provided high-resolution images of breast tissue with sub-centimeter image resolution in less than 2 min, ensuring a quick and efficient process for patients. Clinical results from this prototype showcased its capability to detect centimeter-sized tumors. In further research efforts, magnetic nanoparticles have been incorporated into MT to enhance tumor detection specificity, sensitivity, and accuracy [51,52,53]. This innovation holds promise for improving the overall performance of MT in breast cancer diagnosis.

Another significant advancement was made by Jeon et al. [54], who developed a clinical trial prototype MT system operating within the 3 to 6 GHz range and utilizing a fast-forward solver algorithm. This system was tested on women aged 40 to 68 and demonstrated its ability to detect breast tumors as small as 25 mm. Despite these advancements, there are still limitations to MT. It has relatively low spatial resolution and limited penetration depth compared to other imaging modalities, which can impede the detection of small tumors and restrict its widespread use in everyday clinical settings. Further research is needed to fully evaluate the clinical utility of MT and its integration into routine breast cancer diagnosis and management.

Table 1 compares the recently developed MBI systems for breast cancer detection. MT utilizes changes in the dielectric properties of breast tissues to differentiate between healthy and cancerous tissues. Various MBI algorithms, such as confocal microwave imaging (CMI), tissue-sensing adaptive radar (TSAR), microwave imaging via space-time (MIST), multi-static adaptive (MSA) imaging, and holographic microwave imaging (HMI), are used to create a visual representation of the breast’s internal structures based on the collected microwave signals. This form of image processing involves solving an inverse problem, where the information about the tissue properties is estimated or reconstructed from the measured signals. The reconstructed image can provide insights into the presence and location of abnormal tissue, aiding in the detection of breast cancer. The advantages of MBI include non-invasively reducing patient discomfort and avoiding the risks associated with invasive procedures, providing additional information about the breast tissues compared to traditional methods such as mammography, and not involving ionizing radiation, making it a safer alternative for repeated screenings. However, MBI techniques are still in the research and development stage, with limited clinical availability. The accuracy and sensitivity of MBI methods need further improvement to match or exceed existing those of methods such as mammography. The complexity of image reconstruction algorithms and hardware requirements may hinder their widespread adoption and affordability. Future research and the development of MBI methods are expected to address these limitations for breast cancer detection. Improvements in image resolution, data acquisition speed, and reconstruction algorithms may enable it to become a viable alternative or complementary technique to existing methods. Large-scale clinical trials and studies are needed to validate its performance and ensure its effectiveness in real-world scenarios.

### 4.2. Radar-Based Microwave Breast Imaging Approaches

In 1997, Bridges et al. [119] developed the first radar-based MBI system utilizing UWB microwave signals and an antenna array placed at different locations surrounding the breast to illuminate breast tissues and collect backscattered signals to identify tumors based on the dielectric property difference between normal and tumor tissues.

Radar-based MBI techniques include CMI, TSAR, MIST, MSA, HMI, and time domain data-adaptive (TDDA) imaging. These techniques use various methods to measure and analyze electromagnetic signals to create high-resolution images of target objects. CMI uses a highly focused microwave beam to perform subsurface imaging to reconstruct high-resolution images of target objects. TSAR measures EM signals that penetrate target tissue and analyze and image biological tissues. MIST uses measured radar signals from different locations and times to produce a 3D breast image. MSA uses multiple transmit-receive radar pairs placed strategically for breast screening, while TDDA uses advanced algorithms that analyze the radar signal’s time domain characteristics to perform subsurface target imaging.

Various beamforming algorithms have been applied for breast cancer detection, which are classified into eight categories in Table 2: image reconstruction algorithms, year of study, operating frequency, number of antennas employed, radar-based MI techniques, 2D or 3D image, and a tumor size that can be detected. Table 2 compares radar-based MBI techniques such as CMI, MIST, and TSAR. These methods work by sending low-power microwave signals into breast tissue and detecting the reflected signals. The scattered signals are collected using an array of antennas and processed to generate a 2D or 3D breast image. Radar-based MBI methods play a crucial role in improving the accuracy and reliability of MBI systems for detecting breast abnormalities and offer several advantages over traditional breast imaging modalities such as X-ray mammography. Radar-based MBI methods are safe for frequent examinations. Additionally, microwave signals can penetrate dense breast tissue more effectively, making them suitable for women with higher breast density. Each radar-based MBI technique has advantages and limitations regarding imaging quality and resolution for different breast abnormalities. It is important to note that radar-based MBI methods are still under active research and development. While these techniques show promise in early studies, further validation and refinement are necessary before they can be widely adopted as standard breast cancer detection tools.

Various breast cancer detection systems have been developed, utilizing different technologies and techniques. For example, researchers from the University of Bristol developed a multi-static adaptive system (MAS) consisting of 16 UWB aperture-coupled stacked-patch antennas placed on a hemisphere section to better conform to the shape of the breast. Frenchay Hospital Breast Cancer Center researchers conducted a clinical trial using a radar system consisting of 31 antennas, which was later upgraded to a system with 60 antennas to address issues related to slight patient movements during scans and variations in blood flow and temperature [211].

Hagness et al. [56] developed the contrast-enhanced microwave imaging (CMI) method for breast tumor detection, which could detect small tumors with a diameter of 2 mm using a 2D FDTD solver. However, the FDTD algorithm required significant computational resources and long simulation times. Karamfard et al. [175] proposed time domain synthetic aperture radar (TSAR) imaging for detecting breast cancer, which could detect lesions with a diameter greater than 4 mm. However, the hardware system for TSAR was expensive and caused significant reflections from the skin, presenting a challenge in obtaining clear imaging results.

Bond et al. [63] developed a MIST system consisting of 16 UWB horn antennas for breast cancer imaging, which was later upgraded to improve detection accuracy and could detect small tumors with a diameter as small as 4 mm. These systems demonstrate the potential of microwave-based breast cancer detection and highlight the continuous efforts of researchers to improve the accuracy and effectiveness of these systems for the early detection of breast abnormalities.

Researchers have extensively investigated near- and far-field HMI techniques for detecting biological objects and breast tumors. Elsdon et al. [212,213,214] proposed near-field HMI for biological object detection, which was cost-effective compared to other radar-based imaging techniques. Wang et al. [215,216,217] focused their research on far-field HMI for various biomedical applications, including detecting breast tumors, which requires a shorter data acquisition time than near-field HMI does. In their recent work, the authors introduced a compressive sensing technique to HMI to overcome the challenge of producing high-resolution images with a lower sampling rate.

### 4.3. Deep Learning Approaches for Medical Breast Imaging Applications

In medical breast imaging, machine learning (ML) approaches, including deep learning (DL), convolutional neural networks (CNN), deep neural networks (DNN), and radial basis function neural networks, have been applied for various purposes, such as tumor detection [218], classification [219], and segmentation [220]. Although some of these approaches were validated using only synthetic data, they showed promising results and could be applied to real-world scenarios. For example, Chen et al. [221] utilized a CNN to learn the complex mapping from MR images to dielectric images, which was used as prior information for microwave images. Rekanos et al. [222] developed radial basis function neural networks to estimate the location and size of proliferated marrow inside bone tissue.

In MBI, ML algorithms have been utilized for tumor detection, classification, and segmentation. For example, Shah et al. [223] incorporated learning using a CNN in the second stage of the two-stage approach to improve the spatial resolution of microwave images. Yahya et al. [224] combined wavelet transforms and neural networks to diagnose early breast cancer with 100% success in tumor detection. Li et al. [225] applied a DNN to improve microwave imaging performance, and Khoshdel et al. [226] investigated the feasibility of using DL with U-net to enhance 2D and 3D breast images. The training dataset consisted of 3D contrast-source inversion (CSI) images. The network successfully removed artifacts in CSI reconstructions and improved tumor detectability. Although the network was trained using only synthetic data, it performed exceptionally well with synthetic and experimental data.

Xu et al. [227] developed an end-to-end DL-based method to reconstruct pressure density images directly from sinogram data. They created a TAT-Net to transfer the sinogram domain to the image domain. The TAT-Net reduced the root mean square error to 0.0143 and increased the structural similarity and peak signal-to-noise ratio to 0.988 and 38.64, respectively. These results demonstrate the potential of the TAT-Net in improving image reconstruction quality and facilitating fast quantitative reconstruction.

Tumor segmentation is a critical task in medical breast imaging, and various ML algorithms have been utilized for this purpose. Yang et al. [228] proposed an automatic segmentation model that combines U-Net and level set methods for medical images. Rana et al. [93] applied ML in radar-based imaging for breast lesion detection. Wang [11] proposed a modified AlexNet with transfer learning to automatically detect, classify, and quantify different high-resolution microwave imaging breast images. The proposed transfer learning network achieved 100% accuracy in identifying and classifying HMI images, demonstrating promising applications in microwave breast imaging.

Tumor classification is crucial in determining cancer type and guiding treatment decisions. Mojabi et al. [229] proposed a CNN with the U-Net architecture for classifying breast tissues using tomographic microwave and ultrasound images. Gerazov et al. [219] investigated the feasibility of using a DNN for breast tumor classification and achieved an accuracy of 93.44%. These ML approaches demonstrate great potential in improving breast cancer detection and diagnosis.

DL approaches have demonstrated promising results in improving tumor detection sensitivity and enhancing the quality of microwave images. By rapidly processing large volumes of data, DL-based techniques are valuable in medical imaging applications where efficiency is crucial. Moreover, these approaches can detect and classify tumors automatically, reducing the reliance on manual intervention and minimizing human error. However, it is important to acknowledge the limitations of DL-based microwave imaging approaches. Requiring a substantial amount of training data poses a challenge in medical imaging applications, as acquiring such large datasets can be difficult. Additionally, the interpretability of DL-based methods is a concern in medical contexts where understanding the reasoning behind a decision is crucial. The lack of explainability can hinder the widespread adoption of DL-based approaches in certain medical settings.

Furthermore, DL-based methods demand significant computational resources to train and deploy, making them potentially expensive and limiting their application in resource-constrained environments. These cost considerations can hinder their implementation in certain healthcare institutions or regions. Therefore, further research and validation are necessary to ensure the effectiveness and practicality of these approaches in real-world scenarios.

## 5. Microwave Breast Imaging Systems

A typical MBI system generally includes a microwave signal generator to generate microwave signals, microwave transmitters and microwave detectors, and a computer with an imaging program tool to analyze the measured microwave signals to reconstruct the target breast.

### Microwave Antennas and Antenna Arrays for Breast Cancer Detection

Microwave antennas and antenna arrays are crucial in the MBI system. These antennas emit low-energy microwave signals that can penetrate breast tissue. As these signals interact with the tissue, they undergo scattering and are reflected to the antenna. These scattered signals contain valuable information about the internal structure of the breast tissue, which can be utilized for imaging. Multiple antennas are used in an antenna array configuration to enhance the spatial resolution and image quality of the MBI system. This approach has the potential to improve early detection rates and reduce patient discomfort that may be associated with traditional microwave imaging algorithms. However, the current performance of these antennas, in terms of their radiation capacity, gain, and bandwidth, requires improvement.

Monopole antennas offer a simple design among the different antennas used in the MBI system. They can be easily fabricated using printed circuit board (PCB) technology, making them cost-effective. Slot antennas, on the other hand, provide wideband performance and have a simple and low-cost design. UWB patch antennas are compact and low-profile, making them suitable for integration into wearable breast imaging systems. However, similar to other antenna types, their radiation capacity, gain, and bandwidth performance require improvement.

Regarding clinical trials and prototype testing, several groups have made significant progress in breast imaging. For example, the University of Bristol and Micrima Ltd. (Bristol, UK) conducted clinical tests on their MBI prototype called MARIA, involving 225 patients. They achieved a sensitivity rate of 76% [230]. Umbria Bioengineering Technologies (Rivotorto, Italy) conducted clinical trials on their prototype MammoWave, involving 58 patients, and achieved a sensitivity of 74% [231]. They also reported using support vector machine-based MBI for automatically identifying breast lesions, with an accuracy rate of 91%, a sensitivity rate of 84.4%, and a specificity rate of 97.2% [232]. Mitos Medical Technologies (Istanbul, Turkey) tested their MBI device called SAFE on 54 subjects, achieving a sensitivity rate of 63% [233]. Microwave Vision (MVG) Medical Imaging Department (Paris, France) clinically validated their MBI prototype called Wavelia on 24 patients, successfully distinguishing benign from malignant lesions with an accuracy rate of 88.5% [234].

Recently, researchers have started utilizing new technologies such as MTM [17], MTS [18], and AMC [19] in the development of microwave antennas. For instance, Hamza et al. [16] proposed an MTM microstrip patch antenna with AMC to enhance gain, achieving a gain of 10.61 dBi at 8.6 GHz. Mahmood et al. [21] designed UWB four-element multiple-input and -output (MIMO) wearable antennas to improve detection accuracy. With the advancement of the metasurface, MIMO, and deep learning technologies, new opportunities have emerged for researching and developing UWB MTS antennas to enhance detection accuracy and sensitivity further.

## 6. Challenges and Future Works

In the past few decades, researchers worldwide have extensively studied various breast tumor detection methods, including X-ray mammography, breast ultrasound, breast MRI, and microwave breast imaging. However, each technique faces some challenges. Mammography involves exposure to ionizing radiation, which may pose a risk, especially for women with a high genetic predisposition to breast cancer or those who require frequent screenings. Mammograms can be less effective in detecting tumors in women with dense breast tissue, reducing sensitivity. Compression of the breasts during mammography can cause discomfort and pain for some women. Possible ways to overcome these challenges include developing advanced imaging techniques such as digital breast tomosynthesis, which can provide clearer images and reduce false positives and negatives. The integration of artificial intelligence (AI) algorithms can aid radiologists in interpreting mammograms and lessen the chances of human error. Considering individual risk factors, genetic predispositions, and breast density, personalized screening strategies can improve detection accuracy and limit unnecessary screenings. Research on alternative breast imaging methods, such as ultrasound, MRI, or microwave breast imaging that do not involve ionizing radiation can help mitigate the risks associated with mammography.

The accuracy of breast ultrasound highly relies on the operator’s skills and experience, making it susceptible to inter-operator variability. Ultrasound may be less effective in detecting certain tumor types, particularly smaller lesions or those deep within the breast. Breast ultrasound can result in false-positive findings, leading to additional unnecessary biopsies or interventions. High-quality breast ultrasound machines may not be readily available in all healthcare settings, restricting access for some patients. Possible ways to overcome these challenges include establishing standardized breast ultrasound protocols and ensuring adequate operator training, which can improve consistency and accuracy. Developing AI algorithms that can automatically analyze ultrasound images and assist in detecting abnormalities can reduce operator dependence and enhance sensitivity. Advancements in ultrasound technology, such as shear wave elastography or contrast-enhanced ultrasound, can improve the sensitivity and specificity of breast ultrasound. Research on cost-effective ultrasound equipment and mobile ultrasound devices can improve accessibility and reduce the resource burden associated with breast ultrasound.

Breast MRI can produce false-positive results, leading to unnecessary biopsies or interventions. However, MRI scans can be expensive, time-consuming, often requiring around 30–45 min, and challenging for claustrophobic patients or those with mobility issues. Using contrast agents in breast MRI may pose risks, such as allergic reactions or kidney damage, particularly in patients with pre-existing conditions. Possible ways to overcome these challenges include the development of more affordable MRI machines, streamlined protocols, and reduced scan times to make breast MRI more cost-effective and accessible. Research on innovative MRI techniques, such as diffusion-weighted or dynamic contrast-enhanced MRI, can improve accuracy and reduce false positives. Making MRI scans more comfortable, such as by introducing wider bore machines or open MRI systems, can reduce anxiety and claustrophobia. Investigating safer and more tolerable contrast agents for breast MRI can minimize risks and enhance patient safety.

The electromagnetic properties of human tissues reveal the physiological system’s health status. Non-invasive detection and imaging methods have attracted significant attention. Microwave imaging has the potential to non-invasively produce electromagnetic properties of organs through external controllable excitation, dynamic real-time imaging, and electromagnetic attribute reconstruction. However, existing microwave imaging methods face limitations in terms of imaging speed and resolution, which affect their ability to support the 3D electromagnetic attribute modeling of multi-scale tissues and organs in virtual diagnosis. Researchers have focused on developing 3D MBI techniques using driving mechanisms, imaging, and reconstruction algorithms.

1. Driver: Overcoming the heterogeneity and viscosity of tissues and organs is challenging for imaging systems. Most microwave imaging systems use antenna operating frequencies below 10 GHz as scanning drivers. However, small-sized driving sources face limitations in scattering and diffraction, making them difficult to perform three-dimensional imaging directly. To overcome this, researchers need to develop new strategies for driving mechanisms that can effectively overcome the challenges posed by tissue heterogeneity and viscosity.

2. Imaging: Existing MBI devices use multiple-repeat scanning and sampling, resulting in a long scanning time. Two-dimensional microwave imaging techniques usually take several minutes to produce 2D images, and 3D microwave imaging techniques require an even longer time to create 3D images. Developing rapid scanning and imaging mechanisms is a challenging issue in microwave imaging techniques that needs to be solved. Improving imaging speed while maintaining high-resolution imaging is crucial for efficient and effective microwave imaging.

3. Reconstruction: Microwave propagation within organisms involves multiple parameter variables, and existing reconstruction methods may not apply to multi-scale, an-isotropic tissues or organs. Developing efficient, high-resolution, and sTable 3D microwave imaging algorithms is an ideal solution to achieve fast and high-quality 3D microwave breast images, but it is also challenging. Researchers need to focus on developing advanced reconstruction algorithms that can accurately reconstruct the electromagnetic attributes of multi-scale tissues and organs while handling the complexity of the propagation process.

In future research, improving the low signal-to-noise ratio of 3D microwave imaging is a crucial area of focus since it is challenging due to the multi-scale effect of human organs. Dealing with electromagnetic attribute reconstruction in noise interference and obtaining weak signals are also key challenges. Enhancing the time efficiency of 3D microwave imaging is another important scientific issue that requires attention, as long 3D imaging screening times are a significant issue that hinders its practical clinical application. Comparing and evaluating antennas using standardized metrics can help determine the most effective antenna designs and MBI algorithms. Developing wearable microwave imaging systems and antenna arrays can also improve microwave imaging systems’ spatial resolution and image quality. However, there are still challenges to be addressed before microwave imaging can become a widely adopted clinical tool for breast cancer screening and diagnosis. One challenge is the development of imaging systems that can accurately detect small and early stage tumors. Another challenge is the standardization of imaging protocols and the validation of imaging results through clinical trials and comparative studies with other imaging modalities. Researchers must continue to overcome these challenges and establish the reliability and effectiveness of microwave imaging in clinical practice. Overall, future research should focus on leveraging advancements in technology, imaging techniques, and AI algorithms to improve the accuracy, sensitivity, and accessibility of breast tumor detection methods. Collaboration between researchers, clinicians, and industry stakeholders is crucial to address the current challenges and achieve better breast cancer detection and diagnosis outcomes.

## 7. Conclusions

The successful clinical trials of breast cancer detection using microwave sensing and imaging techniques have shown promising results, suggesting that microwave breast imaging (MBI) has the potential to be an additional or alternative tool to the current standard, X-ray mammography, in detecting early-stage breast cancer. It is crucial to focus on developing microwave imaging algorithms and measurement systems to improve the effectiveness and accuracy of MBI. This paper comprehensively summarized different breast imaging approaches, covering the conventional methods and the dielectric properties of biological tissues, microwave breast imaging techniques, and microwave measurement systems. The advantages of microwave breast imaging techniques were extensively discussed, including the ability to provide safe, non-ionizing radiation imaging, the potential to detect small and soft tissue abnormalities, and the ability to classify malignant and benign breast tumors. However, implementing MBI techniques has challenges that must be addressed. These challenges include variability in breast tissue composition, accurate modeling of complex breast geometries, compensating for patient movement, and achieving optimal resolution. The paper explored possible solutions, including developing sophisticated computational algorithms for the variability in breast composition, using advanced imaging techniques to handle complex breast geometries, implementing motion compensation techniques, and improving sensor arrays to enhance resolution and image quality.

Furthermore, potential areas for future research and improvement in microwave breast imaging techniques were highlighted in this study, including the integration of multiple imaging modalities to enhance diagnostic accuracy, exploration of advanced machine learning techniques for data analysis, investigation of new sensor technologies, and development of personalized imaging protocols for patient care. Overall, the paper provides a comprehensive overview of the recent advancements in microwave imaging and sensing for breast cancer detection. It emphasizes the potential of MBI as a valuable tool in early-stage breast cancer detection. It explores various strategies to enhance the effectiveness and accuracy of microwave breast imaging techniques. Additionally, it identifies areas for further research and improvement to maximize the potential and clinical application of MBI.

## Figures and Tables

**Table 1:** MBI systems for breast cancer detection.

Year	Authors	Frequency (GHz)	Method	Test Method	Image	Antenna	Clinical Trials	Research Findings
1990	Bocquet et al. [55]	2.5–3.5	Radiometry	Human	2D	Multi-probe radiometer	97 patients	The malignant lesions had a radiometric ratio greater than 65%
1998	Hagness et al. [56]	N/A	Confocal microwave imaging (CMI)	Simulation	2D	Monopole antenna	No	Could detect tumors (2 mm in diameter)
1999	Hagness et al. [57]	N/A	CMI	Simulation	3D	UWB bowtie antenna	No	Could detect early stage breast cancer
2000–2001	Bulyshev et al. [58,59]	2, 3.5, and 5	MWT	Simulation	2D and 3D	Helmholtz equation	No	Could detect tumors with a size > 1 cm
2000–2001	Fear et al. [60,61]	N/A	CMI	Simulation	2D and 3D	bowtie antenna	No	Could detect and localize breast tumors in 3D
2003	Li et al. [62]	1–11	Microwave imaging via space-time (MIST)	Phantom	N/A	UWB antennas	No	Could detect very small tumors
2003–2005	Bond et al. [63,64]	N/A	MIST	N/A	N/A	Horn antenna	No	Could detect very small tumors
2004	Dobrowolski et al. [65]	1.5–4.4	Radiometry	Simulation and phantom	2D	Three-band radiometer with mini hypodermic probes	No	Could detect and measure spatial temperature distribution inside a human body
2006	Xie et al. [66]	N/A	Multi-static adaptive (MSA) imaging	Simulation	3D		No	The system showed better resolution and noise rejection capabilities than those of existing methods
2006–2013	Elsdon et al. [67,68,69]	9.4	Holographic microwave imaging (HMI)	Phantom	2D	Transmitting and receiving antenna	No	Could produce real-time images
2010	O’Halloran et al. [70]		MIST	Phantom	2D	horn antenna	No	Could detect tumors (5 mm in diameter)
2010–2012	Son et al. [71,72]	0.5–3	Microwave tomography (MT)	Phantom	2D and 3D	monopole	No	Could detect tumors (5 mm in diameter)
2000–2014	Meaney et al. [73,74,75,76]	1300 MHz	MT	Phantom	2D, 3D	Monopole antenna	500+ patients	Early stage breast cancer detection
2003–2012	Fear et al. [77,78,79]	0.05–15	Tissue sensing adaptive radar (TSAR)	Phantom	2D, 3D	single antenna	8 patients	Could detect and localize tumors with sizes of >4 mm in diameter
2013	Aguilar et al. [80]	1.36–3.03	CMI	Simulation & phantom	3D	slot-loaded patch antenna	No	Miniaturized antennas are suitable candidates as array elements for multi-band microwave breast imaging systems
2013–2018	Wang et al. [81,82]	12.6	HMI	Simulation & phantom	2D	16-element sensor array	No	Could detect small tumors (<5 mm in diameter)
2015	Bucci et al. [83]	2	Magnetic nanoparticle enhanced MWI	Simulation and phantom	2D	Using 50 antennas on each of the eight measurement circles present in the design	No	Allowed the measurement of the differential field arising in an MNP-enhanced way
2016	Medina et al. [84]	2–15	HMI	Phantom	2D	Vivaldi antenna	No	Could detect tumors (>15 mm in diameter)
2008–2016	Klemm et al. [85,86,87,88]	4–10	MSA	Phantom	2D, 3D	16 stacked patch antennas	86 patients	Could detect tumors 4 to 6 mm in diameter
2016	Porter et al. [89]	2–4	Active	Human		Multistatic radar with the 16 sensors	3 patients	Many sources of variability were identified
2017	Song et al. [90]	3.1–10.6	Active	Human	3D	4 × 4 dome antennas	5 patients	Could detect tumor
2017	Yang et al. [91]	4–8.5	Active	Phantom	2D	UWB horn antenna	11 patients	Sensitive to increased tissue
2017	Kuwahara et al. [92]	1–3	MIST	Simulation	3D	stacked patch antennas	2 patients	Data correlation between the measured and calculated data is larger than 0.99
2019	Rana et al. [93]	1–9	Radar-based	Simulation	2D	N/A	clinical trials: normal and malignant tissues	Higher specificity
2019	Sani et al. [15]	1–9	Active	Simulation	2D	one transmitting antenna and one receiving antenna	clinical trials: normal and malignant tissues	The apparatus is safe
2019	Hammouch et al. [94]	3.1–14	CMI	Simulation	2D	Microstrip patch antenna	No	Could detect breast tumor
2019	Islam et al. [95]	2.80–7.00	Radar-based	Simulation and phantom	2D	side-slotted Vivaldi antenna	No	Could detect tumors inside the breast
2019	Srinivasan et al. [96]	2.45	Dielectric substrate	Simulation	2D & 3D	textile wearable antenna	No	Could detect tumors
2019	Soltani et al. [97]	2.45	Microwave-induced thermoacoustic imaging	Simulation	3D	rectangular waveguide	No	Could detect small tumors (5 mm in diameter)
2019	Sheeba et al. [98]	2.4	Active	Simulation and phantom	2D	Flexible soft-wear hexagonal patch antenna	No	tumor as 20.3 A/m^2^ and 19 A/m^2^ and gain as 7.20 and 7.25 dB
2019	Islam et al. [99]	2.7–11.2	Active	Simulation and phantom	2D and 3D	Metasurface Loaded High Gain Antenna	No	Could detect multiple abnormalities inside the breast
2019	Wang [10]	1–4	Multi-frequency HMI	Simulation	2D and 3D	waveguide	No	Could detect small breast lesions with higher accuracy compared to single-frequency HMI
2020	Felício et al. [100]	2–5 GHz	Radar-based	Simulation and phantom	2D, 3D	antipodal Vivaldi antenna	No	Could detect tumors with different positions
2020	Abdollahi et al. [101]	0.8, 0.85, 0.9, and 0.95 GHz	Active	Simulation	2D	Perfect electric conductor (PEC) chamber and 2D transverse magnetic (TM) transceivers	No	The highest AUC of over 0.99
2020	Kumari et al. [102]	8.5	Near-field HMI	Phantom	2D	Vivaldi antenna	No	Minimum size of 4 mm and a maximum depth of 25 mm in the phantom
2020	Ahmed et al. [103]	6.1–12	Radar-based	Simulation and phantom	3D	18 Peano patch antenna	No	Could improve the image resolution
2020	Rahpeima et al. [104]	2.45	MITAI	Simulation	3D		No	Could detect tumors with a radius of 0.25 cm
2020	Miraoui et al. [105]	4	Radar-based ANN	Simulation	2D	Bowtie antennas	No	ANN presented more precision in tumors detection
2020	Cosgun et al. [106]	1.9–2.02	SPION-enhanced MWI	Simulation & phantom	2D	dipole antennas	No	Could detect smaller tumors
2020	Kaur et al. [107]	4.9–10.9	Synthetic aperture radar	Phantom	2D	microstrip antenna	No	The phantom at 5.7 GHz was 0.271 W/kg, and that at 6.5 GHz was 1.115 W/kg for 1 g of body tissue.
2020	Kaur et al. [108]	3.71–11.48	Radar-based	Phantom	3D	Fork-shaped microstrip patch antenna	No	More reflections, less specific absorption rate and more conduction current
2020	Song et al. [109]	3.5–15	Radar-based	Phantom	3D	16-element dome antenna array	1 patient	Improved image quality
2020	Vispa et al. [110]	1–9	Radar-based	Phantom		horn antenna and microstrip monopole antenna	51 breasts	Microwave images of non-healthy breasts had a mean MAX/AVG of approximately 7% greater than those of healthy breasts
2020	Norouzzadeh et al. [111]	1–9	Radar-based system	Simulation	2D	UWB bowtie antennas	2 patients	Cancerous tissues have inhomogeneous dielectric properties
2020	Xiao et al. [112]	6	MWI	Simulation and phantom	2D, 3D	Patch antenna	No	Could accurately find the permittivity
2020	Mehranpour et al. [113]	1.3–6.8	Radar-based system	Phantom	2D	UWB bowtie antenna	No	Could detect tumors with a radius of 7 mm
2020	Carr et al. [114]		Radiometry	Human		ONCOSCAN system	138 patients	41% higher than mammography
2021	Syed et al. [115]	2–5	Distributed antenna system (DAS)	Simulation	2D	Quasi log periodic	No	Two different locations of tumor in the breast phantom were detected
2021	Mahmood et al. [21]	4.8–30	Near-field microwave imaging	Simulation and phantom	2D	Dual-polarized, multiple input–multiple output wearable antenna	No	A maximum radiation efficiency and directive gain of 96% and 5.72 dBi were obtained
2021	Karam et al. [116]	1.5–4.5	Weighted-DAS	phantom	2D	24 antennas arranged into three rings around the breast	BRIGID dataset consists of 7 realistic physical breast models	The contrast is improved as well, with an absolutely negligible computational load compared to that of DAS
2021	Mehranpour et al. [117]	2.5–15	N/A	Simulation and phantom		Low-profile aperture-stacked patch (LP-ASP) antenna	No	Fidelity factor is obtained more at than 90% and 78% in the E-plane and H-plane, respectively, which are satisfactory in the wide-range angles of 90 from the boresight of the designed antenna
2022	Wang [11]	1–12	HMI and Alexnet	Simulation	2D, 3D	Waveguide antenna	No	Could detect and classify tumors
2023	Jamlos et al. [118]	3.1–10.6	N/A	Simulation and phantom	2D	Zero-index metamaterial superstrates UWB antenna	No	MTM antenna consists of 10 × 5 MTM unit cells that recorded a gain of 5.66 dB, having a return loss (S11) of −19.734 dB at a 2.70 GHz frequency

**Table 2:** Radar-based MBI methods for breast cancer detection.

Image Algorithms	Year	Frequency (GHz)	Antenna Number	MBI Method	Image	Tumor Size (mm)	Ref
Skin subtraction algorithm (SSA)	1999	4 & 6	9	FDTD-CMI	3D	4	[120]
SSA	2000	3.6	30	FDTD-CMI	2D	6	[121]
SSA	2001	6	17	FDTD-CMI	2D	2	[122]
SSA	2001	4	10	FDTD-CMI	3D	6	[123]
SSA	2002	4	16	FDTD-CMI	2D	3	[124]
SSA	2003	6	17	FDTD-MIST	2D	2	[63]
SSA	2004	3.2	24	FDTD-CMI	2D	3	[125]
SSA	2004	8	24	FDTD	3D	4	[126]
SSA	2004	6.5	16	FDTD-CMI	2D	2	[127]
SSA	2006	N/A	13	FDTD	2D	2	[128]
Inverse scattering algorithm (ISA)	2002	4	NA	FDTD-CMI	3D	6	[60]
ISA	2003	10	2	FDTD-CMI	2/3D	12	[129]
ISA	2003	0.8	32	MOM	2D	14	[130]
ISA	2017	6	2		3D	4	[131]
Artifact removal algorithms (AR)	2002	6	49	FDTD-MIST	2D	2	[132]
AR	2004	6	49	FDTD-MIST	3D	4	[133]
AR	2005	4	15	FDTD-TSAR	3D	10	[134]
AR	2006	NA	17	FDTD-MIST	2D	2	[135]
AR	2007	5.5	10	FDTD-MIST	3D	2	[136]
AR	2010	7.5	14	FDTD-MIST	2D	5	[70]
AR	2012	6.85	12	FDTD-CMI	3D	2.5	[137]
AR	2013	4.5	21	FDTD	2D	5	[138]
AR	2014	6	50	FDTD-CMI	3D	8	[139]
AR	2015	5	60	FDTD	3D	NA	[140]
AR	2017	6	25	FDTD	3D	15	[141]
Multistatic Artifact Removal (MAR)	2018	0.05–15	30 × 6	FDTD-CMI	3D	15	[142]
AR	2019	0.5–20	N/A	CMI	2D	18 × 9, 12 × 8	[143]
Gauss-Newton iterative algorithm (GNIA)	2003	0.9	16	FEM-CMI	2D	19	[144]
GNIA	2006	NA	5	FDTD-CMI	2D	6	[145]
GNIA	2010	3	40	FDTD	3D	10 & 20	[146]
GNIA	2010	6	5	FDTD	2D	5	[147]
GNIA	2011	1.3	16	FDTD	3D	30	[148]
Delay and sum logarithm (DAS)	2003	NA	17	FDTD	2D	2	[149]
DAS and AR	2005	6	M	FDTD-MIST	2D	2	[64]
DAS	2006	5	72	FDTD-CMI	2D	4	[66]
DAS	2007	5	6	FDTD-CMI	2D	6	[150]
DAS	2007	5	6	FDTD-CMI	2D	10	[151]
DAS	2007	6	16	FDTD	3D	4 & 10	[152]
DAS	2008	6	16	FDTD	3D	7 & 10	[153]
DAS	2010	6	31	FDTD	3D	7	[153]
DAS	2013	6	16	FDTD	3D	3	[154]
DAS	2013	2	16	FDTD	3D	10	[155]
DAS	2014	2	31	FDTD	3D	4	[156]
DAS	2014	4.5	7	FEM	3D	2	[157]
DAS	2015	NA	16	FDTD-CMI	3D	4	[158]
DAS	2015	3	16	FDTD	3D	N/A	[159]
DAS	2015	3.2	31	FDTD	3D	10	[160]
DAS	2015	7.3	9		3D	NA	[161]
DAS	2016	6	20	FDTD-CMI	3D	3 & 5	[162]
DAS	2016	9	1	FDTD-CMI	2D	15	[84]
DAS	2016	4.7	16		3D	3	[163]
DAS	2016	1	16		3D	NA	[164]
Weighted DAS	2021	0.5–10	24	CMI	2D	BRIGID dataset of the National University of Ireland Galway	[116]
DAS and robust capon beamforming (RCB)	2009	6	16	FDTD	3D	4 & 6	[86]
DAS and Multiple Signal Classification	2013	2	31	FDTD	2D	20	[165]
Robust capon beamforming	2011	5	7	FDTD	2D	6	[166]
Delay-multiply-and-sum (DMAS)	2010	7.5	53	FDTD-CMI	2D	5, 10 & 15	[167]
DMAS	2010	3.5	31	FDTD-CMI	3D	7	[168]
DMAS	2011	7.5	20	FDTD-CMI	2D	2.5 & 5	[169]
DMAS	2012	5	36	CMI	2D	2	[170]
DMAS	2012	5	12	FDTD-CMI	2D	2	[171]
DMAS	2015	2.5	16	FDTD-CMI	3D	10	[172]
DMAS	2015	3	16		3D	10	[173]
DMAS	2016	3	16		3D	NA	[174]
DMAS	2017	5	73	FDTD	2D	14	[175]
DMAS	2021	N/A	16	FDTD-CMI	2D	5	[176]
Delay-multiply-and-sum-degree-4 (DMAS-D4)	2022	N/A	N/A	CMI	2D, 3D	N/A	[177]
Double-constrained robust Capon beamforming (DCRCB)	2013	5	9	FDTD	3D	2	[178]
Double constrained robust Capon beamforming (DCRCB)	2015	NA	8	FDTDI-CM	3D	4	[179]
Modified delay-and-sum (MDAS)	2015	NA	16	FDTD-CMI	3D	NA	[180]
Time-of-arrival algorithm (TOA)	2006	6	9	FDTD	2D	4	[181]
TOA	2009	4	128	FDTD	3D	Irregular shaped	[182]
TOA	2011	9.75	22	FDTD-CMI	3D	3	[183]
Time Reversal (TR)	2006	NA	22	FDTD	3D	3	[184]
TR	2007	6	13	FDTD-CMI	2D	10	[185]
TR	2007	9	2	FDTD	2D	2	[186]
TR	2008	8	7	FDTD	2D	4 & 6	[187]
TR	2008	5	16	FDTD	2D	8	[188]
TR	2009	NA	24	FDTD	2D	Irregular shaped	[189]
TR	2011	NA	NA	FDTD	2D	2	[190]
TR	2012	5	29	FDTD	3D	7	[191]
Time-Reversal Multiple Signal Classification (TR-MUSIC)	2016	4	4		3D	NA	[192]
TR-MUSIC	2017	3	115	FDTD	3D	10	[193]
Robust and artifact-resistant (RAR)	2015	6.85	24	FDTD	3D	10	[194]
Inverse Fast Fourier Transform (IFFT)	2005	7	M	FDTD	2D	4	[195]
IFFT	2006	3	16	FDTD	2D	10	[196]
IFFT	2011	4	12	FDTD-CMI	2D	4	[197]
IFFT	2011	4.5	16	FDTD	2D	5	[198]
IFFT	2012	3.5	16	FDTD-CMI	3D	NA	[199]
Matched-filtering algorithm (MFA)	2004	6	2	FEM-CMI	2D	5	[200]
MFA	2006	6	2	FDTD	2D	10	[201]
MFA	2010	5	8	FDTD	3D	N/A	[202]
MFA	2011	N/A	24	FDTD	2D	12	[203]
Generalized likelihood ratio test (GLRT)	2005	6	24	FDTD-CMI	3D	4	[204]
TR and GLRT	2007	6	7	FDTD-CMI	2D	6	[205]
MERIT open-source software	2019	2.5–11	16	Radar-Based Imaging	2D	1.8	[206]
Differential cascode balun low noise amplifier (DCBLNA)	2020	1.5–15.7	N/A	UWB	N/A	highest figure of merit of 3.2	[207]
Hybrid Artifact Suppression (HAS)	2020	2.2–13.5	37	CMI	2D,3D	5	[208]
Contrast source inversion (CSI)	2022	1.38–3.47	10	FDTD-CMI	2D	2	[209]
two-stage rotational clutter suppression (TSR)	2023	3.1–10.6	16	CMI	2D,3D	10	[210]

## Data Availability

Not applicable.
